# ParaKMeans: Implementation of a parallelized K-means algorithm suitable for general laboratory use

**DOI:** 10.1186/1471-2105-9-200

**Published:** 2008-04-16

**Authors:** Piotr Kraj, Ashok Sharma, Nikhil Garge, Robert Podolsky, Richard A McIndoe

**Affiliations:** 1 Center for Biotechnology and Genomic Medicine, Medical College of Georgia, Augusta, GA USA

## Abstract

**Background:**

During the last decade, the use of microarrays to assess the transcriptome of many biological systems has generated an enormous amount of data. A common technique used to organize and analyze microarray data is to perform cluster analysis. While many clustering algorithms have been developed, they all suffer a significant decrease in computational performance as the size of the dataset being analyzed becomes very large. For example, clustering 10000 genes from an experiment containing 200 microarrays can be quite time consuming and challenging on a desktop PC. One solution to the scalability problem of clustering algorithms is to distribute or parallelize the algorithm across multiple computers.

**Results:**

The software described in this paper is a high performance multithreaded application that implements a parallelized version of the K-means Clustering algorithm. Most parallel processing applications are not accessible to the general public and require specialized software libraries (e.g. MPI) and specialized hardware configurations. The parallel nature of the application comes from the use of a web service to perform the distance calculations and cluster assignments. Here we show our parallel implementation provides significant performance gains over a wide range of datasets using as little as seven nodes. The software was written in C# and was designed in a modular fashion to provide both deployment flexibility as well as flexibility in the user interface.

**Conclusion:**

ParaKMeans was designed to provide the general scientific community with an easy and manageable client-server application that can be installed on a wide variety of Windows operating systems.

## Background

Data clustering is a process of partitioning a dataset into separate groups ("clusters") containing "similar" data items based on some distance function and does not require *a priori *knowledge of the groups to which data members belong. Clustering works by maximizing intra-cluster similarities and minimizing inter-cluster similarities. Clustering algorithms are used in various fields such as computer graphics, statistics, data mining and biomedical research. The application of high-throughput technologies, e.g. microarrays, in biomedical research generates an enormous amount of high dimensional data that requires further processing, such as clustering, to reveal biological information.

Clustering algorithms can generally be classified as either hierarchical or partitional. The *k*-means algorithm, introduced by J.B. MacQueen in 1967, is one of the more popular partitioning methods. This algorithm groups data into *k *groups of similar means. The number of groups to be clustered must be defined prior to analysis. The *k*-means algorithm will form *k *distinct nonempty clusters of *m*-dimensional vectors such that each vector is assigned to the cluster with the smallest distance to the cluster's centroid. Several distance metrics can be used, including Euclidean or Manhattan/City-Block distances. A serial *k*-means algorithm has complexity of N*k*R where R is the number of iterations and N is the number of arrays.

Large datasets, such as microarray data, pose new challenges for clustering algorithms. Algorithms with linear complexity, like *k*-means clustering, need to be scaled-up and implemented in a more efficient way to cluster very large data sets. Making the algorithm parallel instead of serial is one potential solution when a sequential clustering algorithm cannot be further optimized. With a parallel algorithm, the computational workload is divided among multiple CPUs and the main memory of all participating computers is utilized to avoid caching operations to the disk, which significantly decrease algorithm execution time. Two general approaches have been attempted at making the *k- *means algorithm parallel: hardware-based solutions (e.g. [1]) and software-based solutions. The use of a reconfigurable array of processors to achieve parallel processing by Tsai et al. provides a good example of a hardware-based solution [1]. A common attempt at a software-based solution involves broadcasting the data to the compute nodes for each iteration [2]. Though this algorithm was faster than the serial version, a major disadvantage is the delay associated with data being sent to the participating nodes during each round of vector assignments. In addition, the number of compute nodes was limited to the number of clusters to be assigned. Another software-based solution for multiprocessor computers is to use a Message-Passing Model, which has been shown to scale up well with the dataset [3-5]. This implementation requires operating system-specific MPI libraries. An example of an MPI implementation can be found at [6]. In addition to the classical *k*-means algorithm, other parallel versions of the variations in the *k*-means algorithm have also been implemented using message passing models. These variations include a parallel version of the bisecting *k*-means algorithm [7] as well as the *k*-means vector quantization (VQ) method [8].

Our focus here is to develop a user-friendly software-based solution that could be utilized by biological researchers. Our solution utilizes a recent modification developed by Zhang et al. that focuses on optimizing the Performance Function [9]. The Performance Function measures the quality of the clusters and, for the *k*-means clustering algorithm, is the sum of the mean-square error of each data point to the cluster centroid [9]. This Performance Function depends only on the global Sufficient Statistic (SS). In this parallel version of the *k*-means algorithm, the global SS are calculated by summing over the SS calculated for each subset of data sent to each node. From the global SS, the new centroids are calculated for each cluster [9]. This transformation makes it possible to implement an efficient parallel processing scheme for the *k*-means algorithm.

## Implementation

### Algorithm

The classical *k*-means clustering algorithm begins by determining *k *initial centroids based on the data to be clustered. These initial centroids can be determined using a number of schemes. However, the most common is a simple random selection of *k *data vectors from the data set. Each remaining data vector (gene expression vector in our case) is assigned to the closest centroid based on a distance metric, commonly Euclidian distance. Once all the data vectors have been assigned to a centroid, a new centroid is calculated for each cluster based on the assigned data vectors. The entire dataset is then reassigned to these new centroids. The algorithm repeats this process of vector assignment-centroid recalculation until the cluster centroids do not change between iterations.

In contrast, the parallel *k*-means clustering algorithm [9] implemented in ParaKMeans follows these steps:

(1) Determine k initial centroids based on the entire dataset. Our program provides three different methods to initialize the centroids (described in detail below).

(2) Divide the whole dataset into subsets (S) equal to the number of compute nodes (M) participating in the algorithm.

(3) Send each data subset (S), the number of clusters (k), and the initial centroid vectors to each compute node for processing.

(4) On each compute node, individual data elements in a subset (S) are assigned to one of *k *clusters based on the shortest distance (using either Euclidean distance or Pearson Correlation) of each element to each cluster's centroid. Note that each data subset (S) may contain elements from all, some or only one cluster and that the calculation of the cluster centroids is based on the entire dataset, not the subsets sent to each node.

(5) Each compute node then calculates the components of the sufficient statistic (SS) for each cluster based on the data subset (S) assigned to each cluster on that node. The components of the SS for each cluster (*k*) on each compute node are the first moment (*FM*), the second moment (*SecM*) and the number of elements (*n*_*c*_) in each cluster:

(1) $$FM_{c}=\sum_{j=1}^{m}\sum_{i=0}^{n_{c}}V_{ij}$$

(2) $$SecM_{c}=\sum_{j=1}^{m}\sum_{i=0}^{n_{c}}V_{ij}^{2},$$

where *c *is the index of the cluster, *i *is the index of gene element, *j *is the index of microarray and *m *is the total number of arrays in the experiment.

(6) The components of the Sufficient Statistics (FM, SecM, and *n*_*c*_) for each cluster, *c*, for each data subset on each compute node are sent back to the master computer to calculate the new global cluster centroids (*gCC*_*c*_) for each cluster.

(3) $$gCC_{c}=\frac{\sum_{p=0}^{M}FM_{cp}}{\sum_{p=0}^{M}n_{cp}},$$

where *p *is the index of child compute node and *c *is the cluster index, and the first and second moments are calculated on each child compute node separately and summarized on the master node.

(7) The Sufficient Statistics are then used to calculate the Performance Function used to measure the quality of the clusters [9]. The global Performance Function is simply the sum of all Performance Functions calculated for each cluster (*Perf*_*c*_).

(4) $$Perf_{c}=SecM_{c}-{\frac{\left(FM_{c}\right)}{n_{c}}}^{2}$$

(8) The new global cluster centroids are sent back to each compute node and replace the previous iterations centroids. The algorithm then loops between steps 4 and 7 above. When the Performance Function reaches a minimum or doesn't change between iterations, the algorithm execution stops and the clustered data are retrieved and collated from the compute nodes.

This parallel implementation of the *k*-means algorithm does not require expensive hardware and the number of compute nodes do not depend on the number of clusters. In fact, any number of inexpensive desktop computers connected by a network can be used. The data partitioning scheme is not restricted and is entirely dependent on the number of compute nodes participating in the algorithm. Additionally, the data subsets (S) are sent only once from the master computer to the compute nodes. Only the data necessary to calculate the sufficient statistics is sent between nodes, dramatically reducing communication latency.

### Metric for Cluster Assignment

ParaKMeans implements two different metrics for assigning a gene (vector) to a cluster. The first is the common Euclidian Distance and the second is Pearson Correlation. For the Pearson Correlation, we use 1-r for the distance calculation. While we can use either metric, all the data generated for this manuscript uses Euclidian Distance.

### Determination of the Initial Centroids

The execution time and cluster quality of *k*-means clustering algorithms are very sensitive to the values of the initial centroids. We provide three different methods to determine the initial cluster centroids:

(1) Random From Data (RFD): *k *randomly selected genes (vectors) are used as the starting centroids. This method is a common method used by most *k*-means algorithm implementations.

(2) Random Initial Assignments (RIA): All genes are randomly assigned to one of the *k *clusters, and the mean of these randomly assigned clusters are used as the starting centroids.

(3) Bisecting K Means (BKM): The initial centroids are calculated using a variation of the Bisecting K Means algorithm [10]. This method works by first randomly selecting one gene (gene0) from the data. The gene (gene1) that is the greatest distance (based on either Euclidean Distance or Pearson Correlation) from that initial gene (gene0) is selected, becoming the first centroid. The gene (gene2) that is the greatest distance from gene1 becomes the second centroid, and the third centroid is the gene that is the farthest distance from both gene1 AND gene2. This process continues until all centroids have been initialized. While this initialization scheme can be time consuming, it provides more stable and consistent clusters.

### Implementation

ParaKMeans is a high performance multithreaded application. We designed ParaKMeans with an easy and manageable client-server application model that can be easily deployed in most laboratories. The system can be deployed on a single computer or across many computers (nodes).

**Figure 1:**
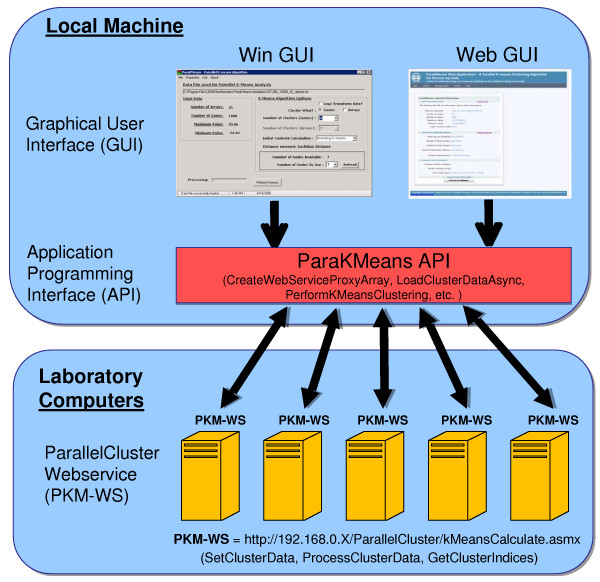
**ParaKMeans software components and deployment strategy**. ParaKMeans has three software components; 1) the graphical user interface (GUI); 2) the application programming interface (API) and 3) the ParallelCluster web service. We provide the GUI in two forms, a windows GUI and a web GUI with the API compiled into each. The GUI is installed on the local machine while the ParallelCluster web services is installed on one or more laboratory computers. Installation of both the GUI and web service is done by double clicking on the .msi installation file and following the installation wizard's instructions.

All the software was written using the .NET Framework v1.1 and C# as the programming language. The application was designed in a modular fashion to provide both deployment flexibility and flexibility in the user interface, and is made of three software components (Figure 1):

**(1) ParallelCluster (The web service)**. A web service is used to perform the distance calculations and cluster assignments, allowing for parallel computation. Web services (servers are not needed to run web services) are a distributed computing technology that makes computing resources (hardware and software) available over the Internet. The technology behind web services is based on common standards of communication, data representation and service description allowing for interoperability between different computers. The web service is responsible for assigning the vectors (e.g., genes) to the centroids and calculating the Sufficient Statistics for that node.

**(2) KMeansMasterComputer (The Main API)**. The main Application Programming Interface (API) is the software component (DLL) that connects to and uses the ParallelCluster web service(s). This library is compiled into and used by the graphical user interfaces. The API provides the methods to load the data, initialize the centroids, partition the data and orchestrate the asynchronous multithreaded connections to the ParallelCluster web services to perform the parallel *k*-means algorithm.

**Figure 2:**
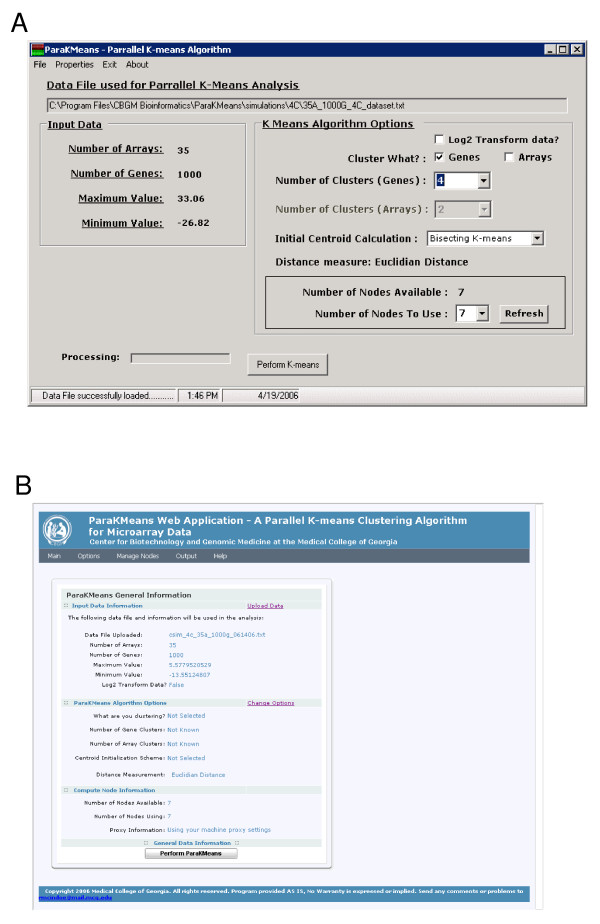
**ParaKMeans user interfaces**. ParaKMeans user interfaces. **A**) Screen capture of the Windows stand alone ParaKMeans application. The interface provides information on the data and program options being used. **B**) Home page of the web based ParaKMeans application, providing an overview of the current data and program options being used.

**(3) ParaKMeans Windows and Web Graphical User Interface (GUI)**. We provide two different graphical user interfaces, a standalone windows GUI and a web based GUI (Figures 1 and 2). The stand-alone GUI can be installed on any Windows machine and provides easy file management, compute node management, program options and a results window for data viewing and saving. The web GUI is an Ajax (Asynchronous JavaScript and XML) enabled website. Ajax is a technique for creating interactive web applications that are more responsive by exchanging small amounts of data with the server behind the scenes. Using Ajax results in only the relevant portions of the web page needing to be posted and reloaded each time the user makes a change. This technique increases the web page's interactivity, speed, and usability. We implemented Ajax using an open source .NET Ajax web control (MagicAjax URL found in Availability and requirements section). The web GUI provides the same functionality as the stand alone program.

ParaKMeans is easily installed using the built-in Windows Installer (MSI files). Essentially, the ParallelCluster web service is installed on each machine that will be a compute node, followed by the GUI being installed on the computer to be directly used by the user. The Windows GUI, the web GUI or both can be installed on each user interface computer. The intended installation is to have one master computer and multiple compute nodes. However, technically, both the GUI and web service could be installed on a single machine. You can simulate multiple compute nodes by adding the IP address of the local machine multiple times. The program will spawn a separate thread for each "node". However, a single machine install will lose any advantage gained by distributing the algorithm (and data) across multiple machines and will impact performance.

### Datasets Used for Analysis

#### Simulated Datasets

We simulated clusters using separate multivariate normal distributions as the basis for each cluster. The center for each cluster was drawn from a uniform distribution with a mean of 0 and a variance between cluster centroids (*σ*^2^_*B*_) was set relative to the variance between means (*σ*^2^_*W*_) within a cluster. A gene specific mean (*μ*_*g*_) was then drawn from a normal distribution with mean equal to the random draw for the cluster (*μ*_*C*_) and the variance equal to *σ*^2^_*W*_. The relative size of the two variances controls the degree of separation between the clusters. For the data we simulated here, we used *σ*^2^_*B *_= 40 and *σ*^2^_*W *_= 0.4. Each array was then simulated using the Cholesky decomposition of the covariance matrix to sample the "error" terms, and a mean vector equal to the vector of *μ*_*g*_. The covariance matrix was defined as follows. The variance between individuals for a gene (*σ*^2^_*g*_) was determined by *σ*^2^_*g *_= *σ*^2^*x/*(*n *- 1), where *x *~Chi-squared(*n *- 1) and *n *is the number of arrays. To ensure that the covariance matrix was positive definite, we set most covariances to 0 with the number of non-zero covariances determined by a Poisson distribution with mean equal to *p*_*C*_(*p*_*C *_- 1)/2 (5*10^-(*log*(*p*)+2)^), where *p *is the number of genes being simulated for the entire array and *p*_*C *_is the number of genes within the cluster. The probability that any given gene pair had some non-zero correlation was then 5*10^-(*log*(*p*)+2)^, which we determined empirically would result in positive-definite covariance matrices. Non-zero covariances were calculated as the product of a correlation that was sampled from a uniform distribution (*r *~U(-1,1)) and the standard deviations for both genes.

We used a combination of conditions for the number of genes (*p *= 100, 500, 1000, 5000, 10000), number of clusters present (*n*_*C *_= 4, 10, 20), and number of arrays (*n *= 35, 100, 200) in the dataset to generate 45 total datasets.

#### Experimental Microarray Data

We also analyzed a dataset that utilized cDNA printed on glass slides to examine expression differences in peripheral blood lymphocytes (PBL) between healthy controls and type 1 diabetic patients [11]. PBL RNA samples were isolated from 59 controls (mean age 13.9 yrs, range 0.5 – 50 yrs) and 35 T1D patients (mean age 17.4 yrs, range 1.3 – 41.2 yrs). To increase the reliability of the data, 2–3 replicate hybridizations were completed for each RNA sample, with the average of the replicates being used in all analyses. Analyses of data for differential expression between controls and patients identified 1195 cDNA clones with differences between the controls and patients. We evaluated the time taken to cluster these data by the various methods and examined the stability of the clusters produced using these data.

### Configuration of Test System Used to Assess ParaKMeans

The length of time to perform the parallel *k*-means clustering algorithm will not only depend on the efficiency of the algorithm, but also the computing hardware used to perform the algorithm. The performance, accuracy and stability of ParaKMeans were evaluated using one master computer with between 1 to 7 compute nodes. For comparisons, we installed Michael Eisen's *Cluster *program [12] on the master computer. We did not compare our program with other parallel software versions of k-means clustering because our goal was to develop a user-friendly version for general laboratory use. The master computer and the seven compute nodes were all identical machines: Dell Poweredge 2650 with Dual 3.06 GHz/512K Cache Xeon Processors and 8.0 GB DDR 266 Mhz RAM.

### Analysis of ParaKMeans Performance, Stability and Accuracy

For all analyses, we used at least twelve replicate runs under each condition to evaluate ParaKMeans, and recorded run time and cluster assignments. Performance was measured using run time, the accuracy of the identified clusters (the extent to which the clusters identified reflected the clusters used in the simulation), and stability of identified clusters (extent of consistency between replicate runs without regard to the actual clusters based on simulation conditions). Run time was log-transformed for all analyses, and factorial analyses of variance (ANOVAs) were used to analyze run time for all comparisons. We observed heterogeneity in the variance of log-transformed run time across the conditions, which we accommodated by fitting a heterogeneous variance model using the Proc Mixed procedure in the SAS statistical system (SAS 2002). We fit multiple models to evaluate which conditions showed the greatest heterogeneity in the variances, and used the model that had the best Aikaike's information criterion (AIC). All significant effects were subsequently examined using Tukey's HSD. We used the adjusted Rand index [13,14] to measure both stability and accuracy. For accuracy, the ARI was calculated for each run relative to the actual cluster identity. The ARI was calculated only between two pairs of replicate runs to measure stability. Comparisons of ARI were based on plots because the ARI showed no variance in several conditions and differences were quite large when they existed.

We first analyzed all 45 datasets using between 1 and 7 nodes to run ParaKMeans with the RFD initialization. A four way ANOVA that included number of nodes, number of clusters, number of genes, and number of arrays was used to examine the differences in run times. This ANOVA model included heterogeneity in the variance among the levels for the number of genes in an array. We next compared ParaKMeans with Eisen's *Cluster *program [12]. For this comparison, we used only those datasets that had 5000 and 10000 genes. A three-way ANOVA that included algorithm (ParaKMeans vs. *Cluster*), number of clusters, and number of genes was used to examine differences in run times. This ANOVA model included heterogeneity in the variance among the levels for the number of genes in an array with these variances also differing between algorithms. We then compared initialization methods for ParaKMeans. For this comparison, we ran ParaKMeans using both 1 and 7 nodes for each initialization method, and we analyzed only those datasets having 100 arrays, 5000 and 10,000 genes with 4 and 20 clusters. We used factorial ANOVA to compare the run times, using a model that included heterogeneity in the variance among the levels for the combination of number of genes and initialization method.

## Results

### Significantly Increased Speedup

The average time taken to cluster each dataset ranged from 0.4 seconds with 7 nodes for a small dataset (e.g. 1000 genes, 35 arrays, 4 clusters) to 24.33 minutes with 1 node for a large complex dataset (10,000 genes, 200 arrays, 20 clusters). The long average time for large complex datasets was reduced to 3.03 minutes using 7 nodes (8 fold decrease in execution time). Execution times were evaluated for ParaKMeans three ways: (1) the effect of the parallel algorithm with multiple computers on time to completion; (2) the point at which adding more compute nodes did not provide any further decrease in time to completion; (3) the effect of the number of arrays, genes or clusters on the execution time of the algorithm.

**Figure 3:**
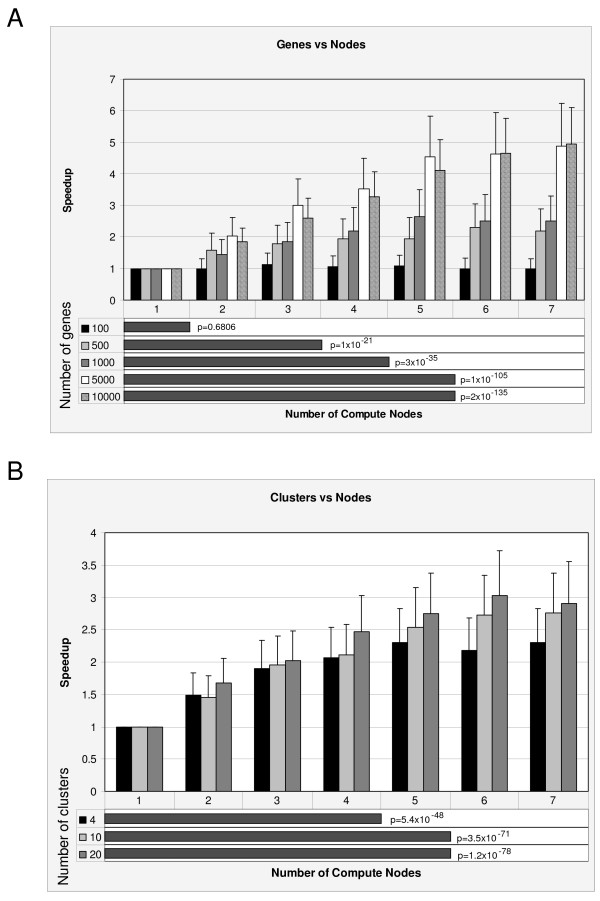
**Detected interactions that affect the time of execution using ParaKMeans**. The column charts plot the speedup (fold increase) relative to a single node configuration versus the number of compute nodes used in the analysis. The bar graphs at the bottom of each plot illustrate the number of compute nodes where one finds statistically significant increases in speed. The p values presented are for tests of differences between the number of compute nodes for a given number of genes or clusters. **A) **The effect of the interaction between the number of genes and number of compute nodes on the speed of execution. **B) **The effect of the interaction between the number of clusters and number of compute nodes on the speed of execution.

Each of these effects was evaluated using ANOVA to assess the interactions between the numbers of genes, arrays, clusters and compute nodes on the length of time to run the program using the simulated datasets (45 separate files, each run 12 times). This analysis detected two significant interactions that could explain the decrease in execution time (Figure 3): an interaction between the number of compute nodes used and the number of genes in the data file (p < 0.0001), and an interaction between the number of clusters being partitioned and the number of compute nodes (p = 0.02). The number of arrays being analyzed (35–200) did have an impact on the time of execution (p < 0.0001), but did not interact with the number of compute nodes, indicating that the decrease in execution time with increasing number of compute nodes was not affected by the number of arrays. We defined speedup as the average time for execution for each test divided by the average time when using only one compute node. All the individual plots of speedup relative to the number of genes, arrays and clusters versus the number of nodes can be found in the online supplement [see Additional files 1, 2, 3].

As now defined, speedup was affected by the number of genes in a data set where no significant speedup was observed for datasets containing 100 genes (Figure 3). Execution time for datasets containing 100 genes did not decrease significantly by increasing the number of compute nodes used, as would be expected. However, datasets with a larger number of genes did show significant speedup, suggesting that datasets containing 500 or more genes will significantly benefit from the parallelization of the *k*-means algorithm. For example, analyses of the 10,000 gene datasets (cross-hatched) using 7 compute nodes had nearly a 5 fold speedup relative to the single node configuration (Figure 3A).

We next asked whether there was a point at which speedup did not increase further by adding more compute nodes. Speedup was affected by the number of genes in a dataset (Figure 3A, bar graph at the bottom of the plot). However, we observed that the increase in speedup obtained by adding compute node became small as the number of compute nodes being compared increased. For example, execution time for analyses of datasets containing 1000 genes did not decrease significantly after 4 compute nodes (meaning 7 nodes are as good as 4 nodes). However, analyses of 10,000 gene datasets showed no further statistical increase in speedup after approximately 5 nodes. We should note that although the results are not statistically significant after 5 compute nodes, the speedup does continue to increase (5 nodes = 4.1 fold, 7 nodes = 4.9 fold). This result is noteworthy since laboratories with as few as 6 computers (1 master and 5 compute nodes) can realize the benefits of parallelization over a broad range of data combinations (i.e. genes/arrays/clusters).

Speedup was also affected by the number of clusters being grouped. As illustrated in Figure 3B (column chart), analyses grouping the data into 4, 10 and 20 clusters significantly benefited from the parallelization of the *k*-means algorithm. The best speedup observed for a given cluster size was a 3 fold increase in speed when *k *= 20 clusters on 6–7 nodes. Again, we found that there was a point at which the number of compute nodes provided no further benefit to speed. One example is that speedup no longer increased beyond using 4 nodes to identify *k *= 4 clusters.

#### ParaKMeans Initialization Schemes

The time analyses described above used the RFD initialization scheme. To test if the initialization scheme could have an impact on the length of time to run ParaKMeans, we recorded the time to completion (n = 12) for ParaKMeans using both 1 and 7 nodes for each initialization method, and we analyzed only those datasets with 100 arrays, 5000 and 10,000 genes with 4 and 20 clusters. The results of the ANOVA analysis indicated that there was a significant difference (p < 0.001) between the initialization methods where the RFD initialization scheme was the fastest followed by BKM and then RIA. Not surprising, for smaller clusters (*k *= 4) the RIA and RFD schemes performed similarly but significantly faster than the BKM (p < 0.001).

### Comparison to Cluster

*Cluster *is a popular gene clustering program written by Michael Eisen [12] and provides the biologist with a variety of clustering techniques. One of these is the *k*-means clustering algorithm. *Cluster *is a Microsoft Windows based program written in C++ and optimized for the Intel architecture. We wanted to determine if our program could perform as well as *Cluster*, since this program is used by many laboratories. The centroid initialization used in *Cluster *is the same as ParaKMeans' RIA initialization scheme. In order to compare the two programs, we ran ParaKMeans using the single compute node configuration and the 'Random Initial Assignment' (RIA) initialization scheme. *Cluster *was installed on the same machine as the ParaKMeans windows application (see configuration of test system above). To compare the time of execution, we used the simulated data files that represented the extremes using the *k*-means algorithm in *Cluster *and the single node/RIA configuration of ParaKMeans. Specifically, we tested the 100 array datasets with 5000 and 10,000 genes for the 4 and 20 cluster datasets. The times for each run were recorded and used in an ANOVA.

**Figure 4:**
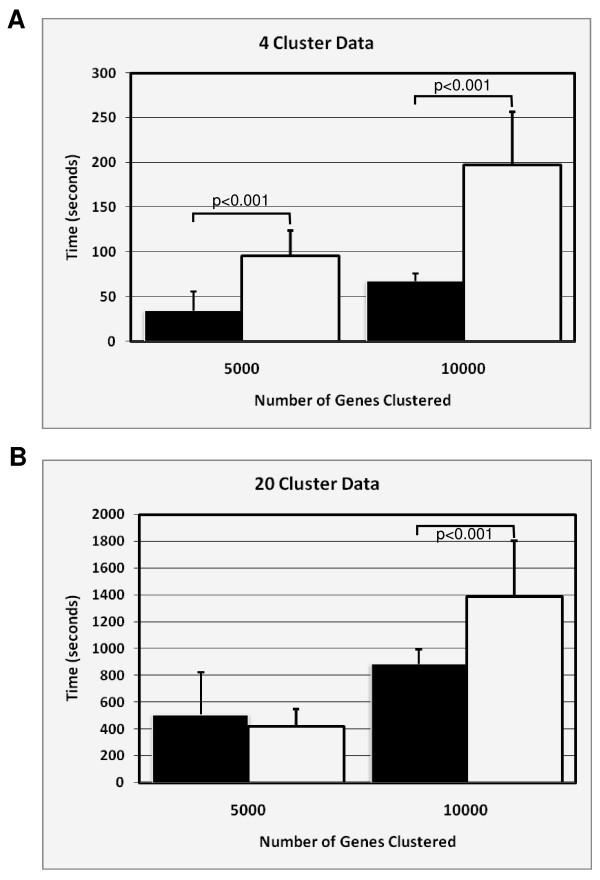
**Time comparison of *Cluster *and ParaKMeans**. ParaKMeans was run using single node/RIA configuration. The calculated p values are presented above the comparisons. **A) **4 cluster data **B) **20 cluster data. White bars = Cluster; black bars = PKM.

ParaKMeans was significantly faster than *Cluster*, overall. With the exception of the 5000 gene/20 cluster dataset (Fig. 4B), ParaKMeans was significantly faster in all combinations of genes and arrays. On average, ParaKMeans was 2.0× faster (range 0.8–2.9) than *Cluster*. This result was surprising considering ParaKMeans must send the data to be clustered to the compute node to be processed, and the computer node is constantly communicating with the master node. This result suggests that the latency involved in the data transfer does not significantly impact the program's performance.

### Evaluation of the Cluster Quality

In order to evaluate the quality of the cluster assignments produced by ParaKMeans, we needed to use a statistic that provided a measure of agreement between the cluster results. A common statistic used to evaluate gene clustering methods is the Rand Index [15]. This statistic indicates the fraction of agreement between two cluster partitions. Agreement can be either pairs of objects that are in the same group in both partitions or in different groups in both partitions. The Rand Index can be between 0 and 1 with 1 indicating perfect agreement. The adjusted Rand Index [13,14] adjusts the score so that the expected value in the case of random partitions is 0. The adjusted Rand Index (ARI) is a popular statistic used to evaluate gene expression clustering algorithms [16,17].

We used the ARI to evaluate both the accuracy and stability of the cluster partitions generated by gene clustering methods. Accuracy was evaluated using the ARI calculated between the resulting cluster partitions and the true partitions. Accuracy is difficult to measure with experimentally derived data since the true clusters are not known. However, the true clusters are known for simulated datasets. Cluster stability was evaluated by calculating the ARI in pairwise comparisons between all resulting cluster partitions, which should assess the consistency of the resulting partitions when the program is run repeatedly on the same dataset. Factors that could affect the accuracy and stability include the number of genes, the number of clusters and the initial centroids used. In assessing the accuracy and stability of ParaKMeans, we analyzed data sets that would be difficult for most programs to cluster accurately. Previous work evaluating the accuracy and stability of gene clustering algorithms used synthetic data with as few as 400 genes [16]. While useful, we wanted to examine how accuracy and stability were affected by much larger datasets. We decided to test the accuracy and stability of ParaKMeans using the 4 and 20 cluster 100 array simulated datasets with 5000 and 10000 genes. As the initialization scheme could affect the results, we ran ParaKMeans using all three initialization methods. We also ran ParaKMeans using a single compute node and seven compute nodes to assess the impact of parallelization on the accuracy. For comparison, we performed a *k-*means cluster of the same datasets using Michael Eisen's *Cluster *program.

Table 1 presents the accuracy and stability results for the 5000 and 10000 gene datasets for both ParaKMeans and *Cluster*. For ParaKMeans, each of the initialization schemes is presented separately. The results for the accuracy and stability when using either 1 or 7 nodes was not statistically different (p = 0.98, data not shown), therefore we combined the results from these analyses (Table 1). As can be seen, the resulting cluster partitions produced by ParaKMeans were significantly more accurate than *Cluster *(p < 0.0001, Table 1). As expected, the number of genes being clustered had an impact on the accuracy of the resulting cluster partitions. This result held for both *Cluster *and ParaKMeans. For example, the median ARI increased from 0.405 for the 5000 gene 4 cluster dataset to 0.594 for the 10000 gene dataset using Cluster, while the median ARI ranges for ParaKMeans were 0.489–0.519 and 0.770–0.896 for the 5000 and 10000 datasets, respectively. In addition, the initialization scheme for ParaKMeans had an effect on the accuracy of the results. Using RIA to initialize the centroids produced results with better accuracy when *k *clusters is small (*k *= 4) and the number of genes is large. For example, the median ARI for PKM-RIA was significantly better then *Cluster*, PKM-RFD and PKM-BKM (10,000 gene, median ARI = 0.896, p < 0.0001). However, as the number of expected clusters increases (*k *= 20) the BKM initialization scheme did significantly better then all others (combined median ARI = 0.545, p < 0.0001)

**Table 1:** Accuracy and stability results using different clustering programs, initialization schemes and number of genes/clusters.

	**Clusters**	**5000 genes**	**10000 genes**	**Combined**
**Accuracy**				
Cluster*	**4**	0.405 (0.404–0.405)	0.594 (0.569–0.604)	0.487 (0.404–0.604)
PKM-RIA*	**4**	0.519 (0.453–0.597)	0.896 (0.896–0.896)	0.747 (0.453–0.896)
PKM-RFD*	**4**	0.519 (0.322–0.519)	0.770 (0.586–0.896)	0.553 (0.322–0.896)
PKM-BKM*	**4**	0.489 (0.489–0.489)	0.770 (0.770–0.770)	0.629 (0.629–0.629)
Cluster*	**20**	0.163 (0.124–0.183)	0.256 (0.196–0.297)	0.190 (0.124–0.297)
PKM-RIA*	**20**	0.231 (0.211–0.461)	0.216 (0.208–0.233)	0.227 (0.208–0.461)
PKM-RFD*	**20**	0.189 (0.178–0.226)	0.210 (0.202–0.252)	0.202 (0.178–0.252)
PKM-BKM*	**20**	0.400 (0.400–0.400	0.691 (0.691–0.691)	0.545 (0.400–0.691)
**Stability**				
Cluster*	**4**	0.439 (0.436–0.442)	0.797 (0.783–0.812)	0.618 (0.436–0.812)
PKM-RIA*	**4**	0.514 (0.435–1.00)	1.00 (1.00–1.00)	1.00 (0.435–1.00)
PKM-RFD*	**4**	0.569 (0.483–1.00)	0.769 (0.586–0.770)	0.711 (0.483–1.00)
PKM-BKM*	**4**	1.00 (1.00–1.00)	1.00 (1.00–1.00)	1.00 (1.00–1.00)
Cluster*	**20**	0.347 (0.321–0.393)	0.492 (0.418–0.907)	0.405 (0.321–0.907)
PKM-RIA*	**20**	0.738 (0.444–0.888)	0.904 (0.682–0.994)	0.788 (0.444–0.994)
PKM-RFD*	**20**	0.594 (0.548–0.643)	0.652 (0.573–0.668)	0.634 (0.548–0.668)
PKM-BKM*	**20**	1.00 (1.00–1.00)	1.00 (1.00–1.00)	1.00 (1.00–1.00)

* – Cluster k-means: Single Node (N = 12), All PKM: 1 and 7 node data (N = 12)

All values are the median adjusted Rand Index with the range of values in parentheses.

Accuracy = cluster results vs. known assignments, Stability = over all agreement between cluster results

PKM = ParaKMeans, RFD = Random From Data, RIA = Random Initial Assignment, BKM = Bissecting K Means, Cluster = Eisen Cluster program.

Interestingly, the resulting cluster partitions produced by ParaKMeans were also more stable when compared to *Cluster*. Similar to the accuracy results, the number of genes, *k *clusters used and the initialization scheme all had an impact on the stability of the resulting cluster partitions. For example, the results using BKM as the initialization scheme produced consistently perfectly stable cluster partitions (ARI = 1.0), while the partitions generated using RIA yielded a median ARI of 0.711 (*Cluster *median ARI = 0.618).

### Real Microarray Data

**Figure 5:**
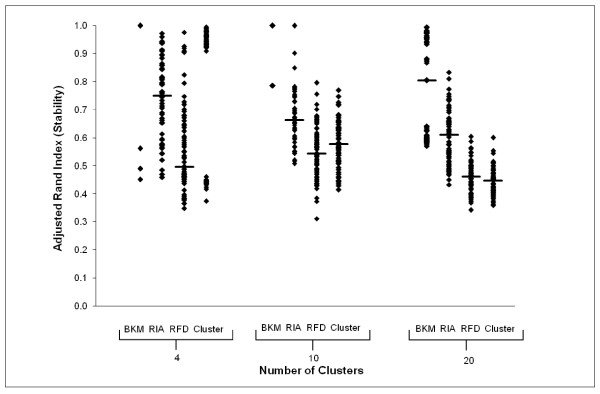
**Stability results for ParaKMeans and Cluster using real microarray data**. ParaKMeans was run using 7 nodes with the initialization scheme indicated on the x-axis. Both ParaKMeans and *Cluster *were run using *k *= 4, 10 and 20 partitions. The median ARI for each analysis is shown using the horizontal line in each plot. Initialization schemes: BKM = Bissecting K Means, RIA = Random Initial Assignment, RFD = Random From Data.

To evaluate how ParaKMeans handles real microarray data, we analyzed the results from a cDNA microarray experiment where we compared gene expression profiles of peripheral blood lymphocytes (PBLs) between healthy individuals and type 1 diabetic patients [11]. This dataset (T1Dset) contained 94 arrays (35 patients/59 controls) and 1195 differentially expressed genes. Unlike the simulated dataset, we did not know the true number of clusters, without which we could not estimate cluster accuracy. However, we could determine the stability of the cluster partitions produced using ParaKMeans and compared these results to what we found using the simulated datasets. We analyzed the T1Dset 12 times each using both ParaKMeans and *Cluster *where *k *= 4, 10 and 20 to assess cluster stability. The resulting cluster partitions were saved for each run and a pairwise ARI was calculated for all 12 runs. Figure 5 is a plot of all the pairwise stability scores (ARI) for each program configuration. As with the simulated dataset, ParaKMeans tended to have a higher median ARI score with the BKM initialization scheme being the most stable. For example, the highest median ARI for the *k *= 20 results was using PKM-BKM (ARI = 0.805) followed by PKM-RIA (ARI = 0.615), PKM-RFD (ARI = 0.461) and *Cluster *(ARI = 0.448). This pattern is the same as we observed with the simulated datasets (BKM>RIA>RFD>*Cluster *when *k *= 20). In addition, only a few PKM-BKM solutions were found for each dataset. For example, when *k *= 10 the PKM-BKM analysis found only 2 solutions (6 runs each gave identical results, ARI = 1.0) while PKM-RIA, PKM-RFD and *Cluster *produced 10, 12, and 12 solutions respectively. This result was similar to the simulated datasets where PKM-BKM gave perfectly stable results (ARI = 1.0, meaning only one solution) and the other algorithms produced multiple solutions.

## Conclusion

This paper presents a parallelized version of the *k*-means clustering algorithm that can be easily deployed by laboratory personnel using most laboratory computers. Often, algorithms that have been converted to run using multiple machines require the use of either specialized hardware or software that may not be accessible to most laboratories. Our goal was to not only produce an efficient parallel *k*-means algorithm, but provide it in an easily deployable manner. Because we use web services, the main requirement for installing the program is that the compute nodes have IIS installed and running. Computers running Windows XP Professional or better will have this installed by default. While we have made every effort to make the software easy to understand and install, users may still have problems. To assist end users, we have created tutorials, troubleshooting guidelines and a full featured help system [see Additional file 2] that provides installation instructions. The help subsystem is provided in two versions, a Windows Help File (.CHM) [see Additional files 4, 5] and help web site [18]. We have also opened the web GUI of ParaKMeans to the public [19].

With respect to the increased speed of execution, Figure 3 clearly demonstrates that ParaKMeans provides a significant increase in algorithm speedup when we use multiple compute nodes. As anticipated, the magnitude of this increase is dependent on the number of clusters being partitioned, the number of genes being clustered and the number of compute nodes participating in the analysis. In addition to the increased speed, ParaKMeans provides consistent and accurate clusters. Because the initialization scheme of the centroids can affect both the accuracy and stability of cluster programs, we provide the user with three alternative strategies to initialize the centroids. Finally, as datasets continue to grow in size, the importance of parallel algorithms becomes more important (Dublin, 2007).

## Availability and Requirements

**Project name: **ParaKMeans

Project home page:



**Online Help: **

**Web version open to the public: **

**Operating system(s): **Windows XP Professional or greater

**Programming language: **C#

**Other requirements: **.NET 1.1 or higher

**License: **Open Software License v3.0, freeware.

**Any restrictions to use by non-academics: **none

**MagicAjax:**

## Abbreviations

PKM: ParaKMeans; RFD: Random From Data; RI: Random Initial Assignment; BKM: Bissecting K-Means; ARI: Adjusted Rand Index.

## Authors' contributions

RAM conceived the software and participated in the evaluation, analysis, design and writing of the software and manuscript. PK participated in writing the software and draft version of the manuscript. RB and NG participated in the design of the study, development of the simulated datasets and statistical analysis. AS participated in the evaluation and analysis of the performance of the software. All authors read and approved the final manuscript.

## Supplementary Material

Additional file 1 Speedup results for the four cluster simulated data using 35, 100 and 200 arrays. For each plot, the y-axis is the speedup value and the x-axis is the number of nodes used to run ParaKMeans. Each line is a different number of genes clustered in that dataset. Click here for file

Additional file 2 Speedup results for the ten cluster simulated data using 35, 100 and 200 arrays. For each plot, the y-axis is the speedup value and the x-axis is the number of nodes used to run ParaKMeans. Each line is a different number of genes clustered in that dataset. Click here for file

Additional file 3 Speedup results for the twenty cluster simulated data using 35, 100 and 200 arrays. For each plot, the y-axis is the speedup value and the x-axis is the number of nodes used to run ParaKMeans. Each line is a different number of genes clustered in that dataset. Click here for file

Additional file 4 ParaKMeans Help system. The Windows Help file contains a description of the program, installation instructions, tutorials and the API documentation. Click here for file

Additional file 5 ParaKMeans windows help file. This file is a windows help file (.chm) that provides a more detailed overview of the software, installation instructions, program tutorials, the ParaKMeans API and troubleshooting help. Click here for file
