# Supplementary material for: ParaKMeans: Implementation of a parallelized K-means algorithm suitable for general laboratory use
Source: BMC Bioinformatics. 2008 Apr 16;9:200. doi: 10.1186/1471-2105-9-200 (PMC2375128; doi:10.1186/1471-2105-9-200)
Supplement: Additional file 1 — Speedup results for the four cluster simulated data using 35, 100 and 200 arrays. For each plot, the y-axis is the speedup value and the x-axis is the number of nodes used to run ParaKMeans. Each line is a different number of genes clustered in that dataset. [file 1471-2105-9-200-S1.ppt]

## Slide 1
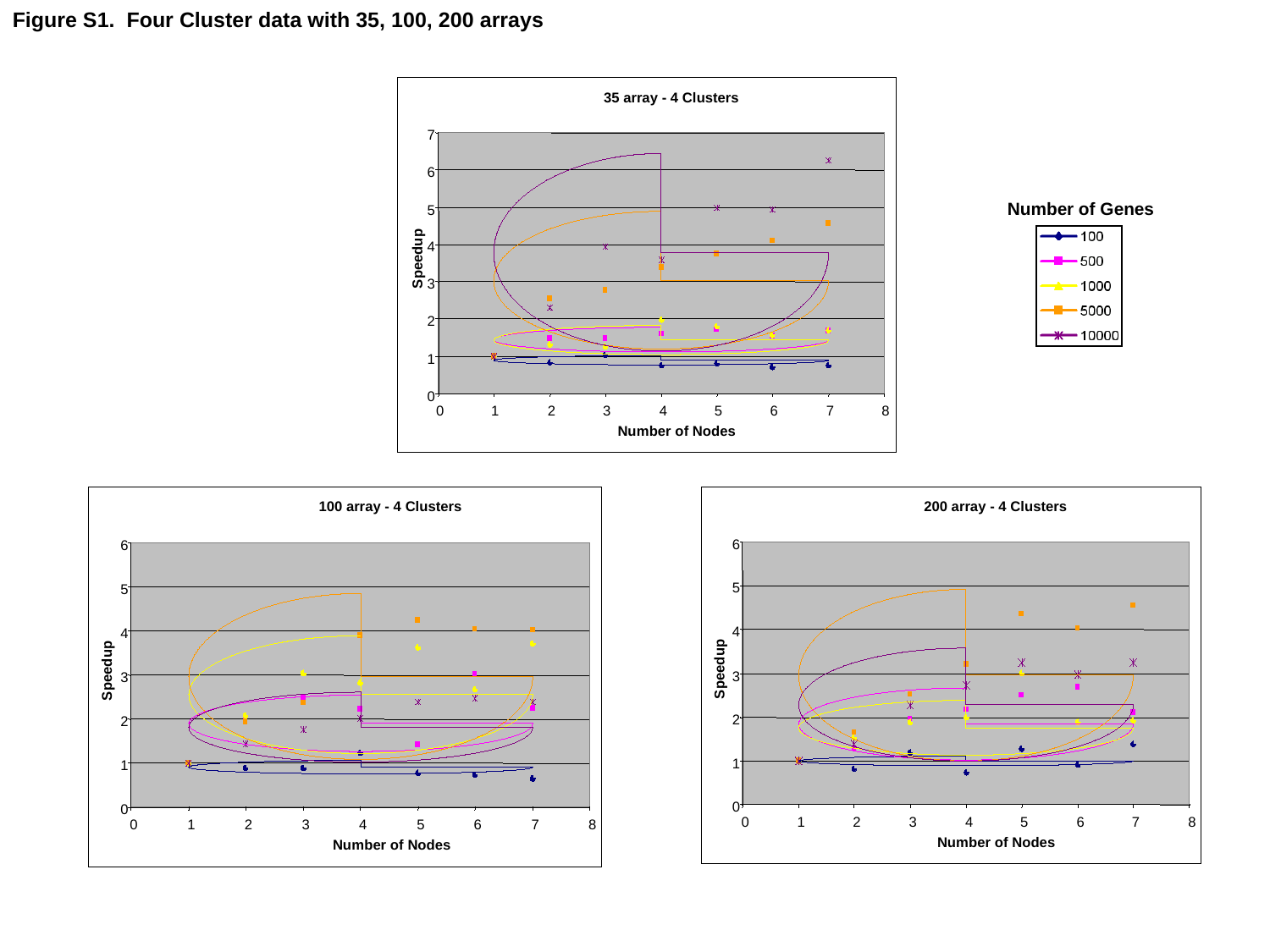

Figure S1. Four Cluster data with 35, 100, 200 arrays
35 array - 4 Clusters
7
6
5
4
Speedup
3
2
1
0
0
1
2
3
4
5
6
7
8
Number of Nodes
Number of Genes
100 array - 4 Clusters
6
5
4
Speedup
3
2
1
0
0
1
2
3
4
5
6
7
8
Number of Nodes
200 array - 4 Clusters
6
5
4
Speedup
3
2
1
0
0
1
2
3
4
5
6
7
8
Number of Nodes
