# Supplementary figures and images for: ParaKMeans: Implementation of a parallelized K-means algorithm suitable for general laboratory use
Source: BMC Bioinformatics. 2008 Apr 16;9:200. doi: 10.1186/1471-2105-9-200 (PMC2375128; doi:10.1186/1471-2105-9-200)

## Slide 1
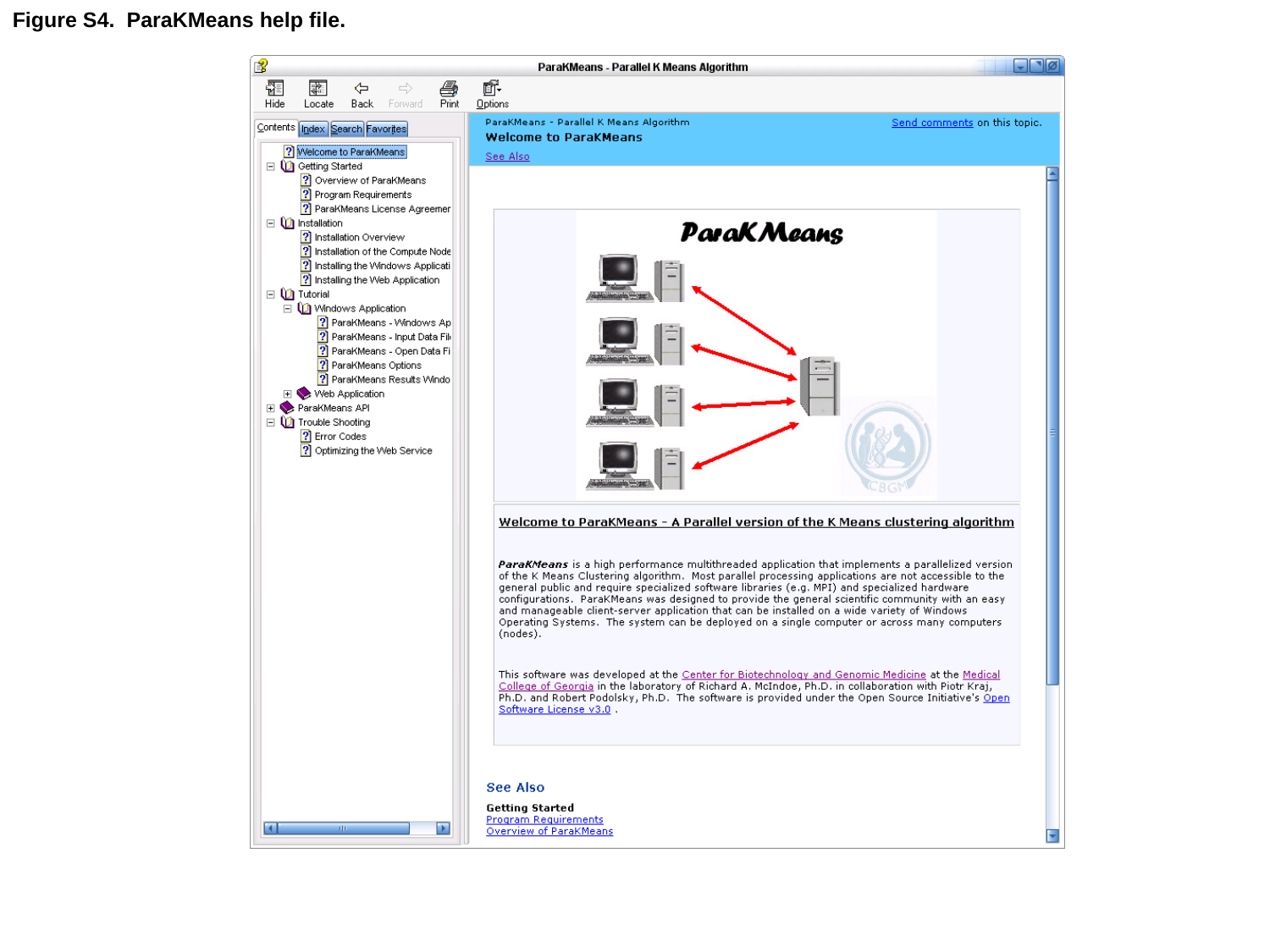

Figure S4. ParaKMeans help file.

Supplement: Additional file 4 — ParaKMeans Help system. The Windows Help file contains a description of the program, installation instructions, tutorials and the API documentation. [file 1471-2105-9-200-S4.ppt]
